# Impact of Safety Warning on Domperidone Prescribing for Geriatric Patients in South Korea: Analysis of National Insurance Claim Data

**DOI:** 10.3390/ijerph16162985

**Published:** 2019-08-20

**Authors:** Kiyon Rhew, Nayoung Han, Jung Mi Oh

**Affiliations:** 1 College of Pharmacy, Seoul National University, Seoul 08826, Korea; 2 College of Pharmacy, Dongduk Women’s University, Seoul 02748, Korea; 3 Research Institute of Pharmaceutical Sciences, Seoul National University, Seoul 08826, Korea

**Keywords:** domperidone, elderly patients, QT prolongation, safety, prescription

## Abstract

Domperidone is a dopamine antagonist used for the symptomatic management of nausea and vomiting. Many countries banned or add a black box warning due to an increased risk of serious adverse cardiac effects. In 2014, the Korea Ministry of Food and Drug Safety also released a safety warning to carefully consider adverse cardiac effects when prescribing domperidone. Therefore, we conducted this study to analyze the impact of the safety warning on domperidone prescribing. This study included patients 65 years or older who used national health insurance services in the years 2011 and 2016, using the national patient sample dataset in South Korea. We analyzed the characteristics of domperidone prescribing and compared on pre- and post-safety warning. Prescribing frequency of domperidone was significantly reduced from 603,962 cases in 2011 to 24,623 cases in 2016. In 2011, 53,272 (8.8%) prescriptions were for greater than 30 mg/day, whereas only 200 (0.8%) prescriptions were in 2016. The number of patients with one or more comorbidities and electrocardiogram monitoring showed positive changes after the safety warning. In conclusion, after the 2014 safety letter was issued, domperidone was more safely prescribed in various aspects in elderly patients, including frequency of prescribing, maximum daily dose, and duration of continuous prescription.

## 1. Introduction

Domperidone is a drug that blocks peripheral dopamine D2 receptors. This drug is typically used to manage symptoms of upper gastrointestinal (GI) motility disorders, including diabetic gastroparesis, nausea, and vomiting. Importantly, domperidone does not cross the blood–brain barrier [1,2], limiting the risk of adverse drug reactions (ADRs), such as akathisia, Parkinsonism, or tardive dyskinesia [3,4]. However, several studies have reported that domperidone increases the risk of cardiac ADRs or sudden death at high doses or in high-risk patients [5,6,7]. Specifically, domperidone may increase the risk of ventricular arrhythmia and sudden death by inhibiting hERG (human ether-a-go-go related gene) channels, leading to QT prolongation [8]. Furthermore, the risk of domperidone-induced cardiac ADRs is significantly higher in elderly patients (>60 years old) compared to younger adults [7]. Elderly people generally have a higher prevalence of arrhythmia or heart failure and many suffer from multiple medical problems, including heart, kidney, and/or liver dysfunction [9,10]. Elderly patients also often take multiple medications, which may also increase the risk of cardiac ADRs and QT prolongation through hazardous drug-drug interactions [11,12,13,14]. Therefore, domperidone use in the elderly population requires careful attention and close monitoring.

After the issues associated with domperidone use became known, many countries acted to either withdraw domperidone from the market or add a black box warning to its labelling [15,16,17]. In 2014, the Ministry of Food and Drug Safety (MFDS, Korean regulatory agency) of the Republic of Korea issued a ‘safety warning letter’ regarding domperidone use [18]. This letter referred to the risk of cardiac side effects such as ventricular arrhythmia, Torsade de Pointes, and QT prolongation. In addition, the Drug Utilization Review System of Korea sends an alert when domperidone is prescribed for patients older than 65. They added some limitations that domperidone should only be used to alleviate symptoms of nausea or vomiting and be administered at a dose of 10 mg three times a day or less for a maximum of one week. The regulatory actions also included a caution to healthcare professionals that co-prescribing medications associated with QT prolongation or Cytochrome P450 3A4 (CYP3A4) inhibition should be contraindicated. Following the issuance of these actions, the label for domperidone now also includes a contraindication in patients with moderate to severe liver failure, QT prolongation, electrolyte disorders (hypokalemia, hypercalcemia, or hypomagnesaemia), or heart failure. A precaution was also added for domperidone administration in elderly patients and patients with cardiac disease except for heart, renal, or mild liver failure. However, how this safety letter affected the rate of domperidone prescription in elderly patients is unknown.

In this study, we sought to analyze the characteristics of elderly patients who were prescribed domperidone and to evaluate how this regulatory action affected aspects of domperidone prescription, including indication, daily dose, and maximum continuous prescribing duration.

## 2. Subjects and Methods

### 2.1. Data Sources and Study Subjects

In the Republic of Korea, the National Health Insurance (NHI) is a universal health coverage system that covers approximately 100% of Korean residents [19]. The NHI system operates as a categorization of the national health insurance program, medical aid program, and veteran’s insurance program. The medical aid program is a kind of public assistance system that supports self-help by ensuring a minimum livelihood and providing health care for low-income households. All patient health-related information is then reported to the Health Insurance Review Agency (HIRA).

The HIRA claims data contains 46 million patients each year and accounts for 90% of the total population in Korea [20]. However, given the complexity and large volume of this data, its use is typically restricted to researchers. To resolve these limitations and increase the efficiency of data use, the HIRA developed patient sample data that passed validity tests performed by five different institutions [20]. This sample dataset comprised 10% inpatients and 90% outpatients. Because the sample sizes were carefully calculated and extracted to improve representation in terms of socio-demographic characteristics and medical information, including diagnosis and prescription drugs, the results of this study can be applied to the entire elderly population in South Korea. Here, we used a dataset of HIRA claims data comprised of randomized patients over 65 who used the National Health System at least once a year (HIRA-APS). This data set passed validity tests performed by five different institutions and included approximately one million patients per year over the age of 65 (~20%) [20]. To compare data before and after the 2014 regulatory action was issued, we used national aged patient sample data from 2011 and 2016 (HIRA-APS-2011 and HIRA-APS-2016).

We considered users who were prescribed domperidone at least once during the indicated years. Demographic analysis included age, gender, and insurance type. Age was classified into 5-year groups starting at 65, and patients were categorized according to their insurance type including health insurance, medical aid, and veteran’s. This study was approved by institution review board (IRB) of Dongduk Women’s University (IRB No. DDWU 1711-01).

### 2.2. Analysis of Domperidone Prescription

Indications for domperidone use and patient comorbidities were analyzed using the Korean Standard Classification of Diseases (KCD)-6 coding for the 2011 dataset and the KCD-7 for 2016 data. Among the patients who were prescribed domperidone, we assessed patient comorbidities including hepatic disease (B15-19, K70-77), renal disease (N00-08, N11, N18-19, N25, or N28), cardiac arrhythmia (I44-49), and Parkinson’s disease (G20, G21). The maximum dose was categorized based on the highest daily dose during that one-year period as follows: ≤30 mg/day, between >30 mg/day and ≤40 mg/day, and >40 mg/day. The maximum duration of consecutive prescriptions was calculated as the total duration of continuous prescription over one year. Because there is no medication refill system in Korea, the maximum continuous duration was calculated using each prescribing date. For example, if a patient was prescribed domperidone for 3 days and then received a 5-day prescription 3 days later, the maximum duration was calculated to be 8 days. The maximum duration of domperidone use was categorized into two groups with the cut-off at 7 days.

### 2.3. Analysis of Co-Prescribed Medications

Co-prescribed medications were defined as medications prescribed concurrently with domperidone. Medications that have been reported to prolong the QT interval or to interact with domperidone by inhibiting CYP3A4 enzyme activity were identified in Supplement Table S1. We extracted these medication lists from domperidone IND packet by US FDA (food and drug administration) and domperidone labeling by MFDS [21,22]. MFDS uses 4-digit numbering codes to identify medication components, and HIRA provides this information in the dataset. We used this 4-digit number to identify the medication. We analyzed the frequency of co-prescriptions of these drugs categorized by ATC code and compared changes in the use of hazardous co-medications between the 2 years.

### 2.4. Performance of Electrocardiogram (ECG) Monitoring before or after Domperidone Use

The risk of QT prolongation may be elevated in patients co-prescribed drugs that interact with domperidone. Thus, we also analyzed whether ECG monitoring was performed before or after domperidone use.

### 2.5. Statistical Analysis

Descriptive statistics for population characteristics were used. To analyze differences in the prescribing patterns between 2011 and 2016, we performed a logistic regression and reported these data as univariate odds ratios (ORs) and 95% confidence intervals (CIs), with a two-sided *p*-value of 0.05 considered statistically significant. All analyses were performed using SAS statistical software (SAS version 9.4, SAS Institute, Cary, NC, USA).

## 3. Results

### 3.1. Patient Characteristics

For the 2011 sample data, 1,073,183 elderly patients were included, of which 434,540 (40.5%) were male and the mean age was 73.63 (standard deviation (SD) 6.58). The 2016 data included a total of 1,327,455 patients, of which 556,699 (41.9%) were male and the mean age was 74.11 (SD 6.72). In 2011, 169,249 patients (15.8%) were prescribed domperidone, of which 111,847 (66.1%) were female. When subdivided into five-year intervals, patients older than or equal to 75 was the age group that were most frequently prescribed domperidone (N = 59,991, 35.4%). In 2016, 12,093 patients (0.9%) were prescribed domperidone, of which 7676 (63.5%) were female and 51.6% were older than or equal to 75. In both years, female patients were prescribed domperidone more frequently than males. Furthermore, more patients under medical aid or veteran’s coverage were prescribed domperidone in 2016 than in 2011 (Table 1). Importantly, the proportion of domperidone prescriptions in the overall geriatric population significantly decreased from 2011 to 2016.

### 3.2. Analysing Domperidone Prescription Patterns

The number of domperidone prescriptions was 603,962 in 2011 compared to 24,623 prescriptions in 2016, which represents a 96% reduction over this five-year period (Table 1). In both years, the most common indications for domperidone use were gastritis and duodenitis, corresponding to 311,879 and 10,591 prescriptions, respectively. In 2011, functional dyspepsia accounted for 103,644 cases, while in 2016, duodenal ulcer was the second most frequent indication, accounting for 4648 cases. The numbers of prescriptions that were not associated with GI diseases or whose indications were unclear were 136,765 (22.6%) in 2011 and 8671 (35.2%) in 2016. Thus, between the two years, the absolute number of prescriptions not associated with GI indications decreased but the proportion increased.

In 2011, 60,881 (36.0%) of domperidone users had one or more comorbidities, including cardiac, renal, hepatic diseases, or Parkinson’s disease, compared to 2525 (20.9%) patients in 2016. Thus, the risk of comorbidities in prescribed patients decreased between 2011 and 2016 in general. In both years, hepatic disease was the most common comorbidity. In 2011, about 3.8% of patients prescribed domperidone had arrhythmia as a comorbidity relative to only 1.6% in 2016. The ORs of Parkinson’s disease, however, was increased in 2016 compared to 2011 (Table 2).

The maximum daily dose of domperidone also significantly differed between 2011 and 2016. In 2011, 550,690 (91.2%) prescriptions were for a daily dose of domperidone that was ≤30 mg/day, while 11,556 (1.9%) prescriptions were for >30 mg/day and ≤40 mg/day, and 41,716 (6.9%) were for >40 mg/day. In 2016, only 98 cases (0.4%) were for >30 mg/day and ≤40 mg/day, while 102 cases (0.4%) were for >40 mg/day. Thus, compared to 2011, the number of prescriptions for domperidone doses >30 mg/day was significantly lower in 2016 (Table 2).

Finally, comparing the maximum continuous duration of domperidone revealed that in 2011, 64,425 patients (38.1%) were prescribed for more than seven days compared to 5221 patients (43.2%) in 2016 (Table 2). Thus, the total number of patients prescribed domperidone for more than seven days significantly decreased in 2016, but the percentage of patients remained high.

### 3.3. Analysis of Co-Prescribed Medications

We next evaluated the number of cases involving co-prescribed medications that may interact with domperidone and prolong the QT interval. Importantly, the number of cases involving co-prescribed medications that may prolong the QT interval significantly decreased from 2011 to 2016 (153,745 in 2011 vs. 10,664 in 2016) (Table 3). Furthermore, the number of cases involving co-prescribed drugs that may interact with domperidone was 165,838 (27.46%) in 2011 compared to 10,816 cases (43.9%) in 2016. In 2011, drugs for acid-related disorders (A02) were the most frequently co-prescribed with 96,031 cases (15.9%), followed by diuretics (C03) with 28,225 cases (4.7%), and antibacterials for systemic use (J01) with 17,080 cases (2.8%). In 2016, 4085 cases involved co-prescribed diuretics (16.6%), followed by 2811 cases (11.4%) of drugs for acid-related disorders (A02), and 2470 cases (10.0%) of psychoanaleptics (N06) (Table 4). Comparative analysis of the frequency of co-prescribed drugs revealed that the absolute number of prescriptions significantly declined between 2011 and 2016 despite the relative proportion increasing.

### 3.4. Efforts to Monitor Cardiac Events after Domperidone Use

In our population, only 3.7% (N = 6263) of patients underwent ECG monitoring before or after domperidone administration in 2011 compared to 48.4% (N = 5852) in 2016 (data not shown).

## 4. Discussion

In this study, we demonstrated that after the MFDS issued the safety letter and regulatory actions in 2014, the number of domperidone prescriptions for elderly patients markedly decreased. To our knowledge, this study was the first to assess the impact of regulatory actions for overall prescribing pattern changes including number of prescriptions, daily dose, continuous treatment duration, indication, co-prescribing medication, patients’ comorbidities, and safety monitoring.

Our data revealed that although the domperidone prescription rate decreased between 2011 and 2016, more female patients and patients older than 75 received domperidone in 2016. In both years, domperidone was prescribed most frequently for symptoms of gastritis and duodenitis. This suggests that the trends of domperidone prescription are associated with the high prevalence of GI diseases in women and/or older patients, as demonstrated by previous studies [23,24]. The elderly populations also generally have reduced organ functions, including the kidney, heart, and liver, and have more comorbidities than younger patients [25,26]. Because domperidone is metabolized in the liver and kidneys, decreased organ functioning may impair drug clearance. A previous study reported that clearance of metoclopramide (similar to domperidone) was reduced by about half in patients with severe liver disease, thereby increasing drug exposure [27]. Thus, the cardiac ADRs associated with high domperidone exposure can be especially dangerous in elderly patients suffering multiple organ dysfunction. In this study, ORs of Parkinson’s disease as comorbidity of patients prescribed domperidone increased. Domperidone has benefit for ADRs because it does not cross the blood–brain barrier. On the other side, a recent study from the UK found that elderly patients using domperidone had a higher prevalence of Parkinson’s disease, nearly doubling all-cause mortality [28,29]. However, it is still important to treat in Parkinson’s disease patients with dopamine agonists, nausea and vomiting are important adverse effects of those. Therefore, domperidone use in elderly patients must be prescribed only when necessary and careful monitoring is needed for a better benefit–risk profile [30].

We also observed decreases in the maximum daily dose, maximum continuous treatment duration, and the number of cases involving co-prescribed medications that could increase the risk of cardiac ADRs from 2011 to 2016. These changes may be due to increased concern from prescribers regarding domperidone-induced cardiac ADRs in geriatric patients. Our results were similar to those in previous studies from the UK and Canada. In the UK, following an adjusted recommendation from the Medicines and Healthcare Regulatory Agency in 2014, the rate of domperidone prescription decreased by about 70% and the number of patients prescribed with doses higher than recommended decreased from 20/64 to 3/19 patients [31]. Here, we report a more dramatic decrease in the domperidone prescription rate compared to this previous study, as we observed that the rate decreased from 15.7% of total elderly patients in 2011 to less than 1% in 2016. This discrepancy may result from the different comparison periods between the studies. Specifically, the UK study analyzed differences over a seven-month period, while our study compared prescription changes over a five-year period. With this longer time period, more physicians could have noticed and heeded the regulatory actions. In another comparative study of domperidone use in a Canadian tertiary care center, the rate of domperidone initiation in the hospital decreased from 71.4% to 39.4% after a safety warning was issued by Health Canada [32]. This warning reduced domperidone use for non-proven indications, prescriptions for doses >30 mg/day, and for patients older than 60. This warning also increased the frequency of ECG performance or electrolyte monitoring before and after domperidone use.

Importantly, monitoring ADR after domperidone use is critical for prevention, particularly in the elderly. Although such monitoring can be done using typical measurements of the QT interval, experts suggest that it is best to measure the QT interval using 12-lead ECG during peak plasma concentrations of a QT-prolonging medication [33]. Despite this, ECG QT interval measurements are not routinely performed in the clinic. One European study suggested that despite the media attention regarding domperidone-induced deaths, the rate of ECG measurements has not significantly changed and only 10% of patients prescribed domperidone receive ECG tests [34]. However, in our study, the rate of ECG monitoring in patients prescribed domperidone increased from 2011 to 2016. Although the absolute number did not increase, 48.4% of patients underwent ECG monitoring in 2016. This suggests that the proportion of patients who underwent ECG monitoring increased because the number of patients prescribed domperidone decreased.

Importantly, the risk of cardiac ADRs increases when QT-prolonging agents or CYP3A4 enzyme inhibitors are used concomitantly with domperidone [35,36]. In a recent study, the rates of concurrent prescriptions involving domperidone and one or more macrolide antibiotics, selective serotonin reuptake inhibitors, or anti-arrhythmic agents did not change or slightly increased [37]. In our study, the number of domperidone prescriptions and number of co-prescriptions involving potentially interacting medications greatly reduced from 2011 to 2016. The co-prescribing rate, especially for relatively acute symptoms or diseases (e.g., antacids and antihistamines), also decreased. Furthermore, the rates of concomitant use of antibacterials (2.8%), psychoanaleptics (2.7%), and drugs for cardiac disease (<0.1%) were low in 2011. These are positive results regarding drug utilization in elderly patients who are vulnerable to ADR. However, in 2016, these rates increased to 4.5%, 10.0%, and 0.6%, respectively. Importantly, because drugs containing domperidone can still be purchased over-the-counter in Korea, prescribers must be cautious when prescribing drugs that can interact with domperidone and must carefully monitor cardiac ADR risk.

This study had several limitations. First, we did not directly confirm the effect of domperidone use on the incidence of cardiac ADRs. We analyzed outcomes of implementation of regulatory actions on domperidone use. Therefore, we could not determine how much each risk factor (dose, duration, concurrent medication, etc.) contributed to cardiac side effects. Second, we only analyzed the number of ECG measurements, but we did not include why the physicians did or did not monitor. This is important because in some cases, ECG monitoring may not be considered necessary or the physician was not concerned because relevant information was not on the label. Alternatively, physicians may have monitored an ECG if a patient reported with arrhythmia or other cardiac symptoms. Third, we could not determine which alternative antiemetic drugs were prescribed or no longer prescribed after the safety warning on domperidone. Lastly, the number of domperidone prescriptions significantly differed between the two years. This limited the ability to directly compare the proportions of each risk factor between the two years. Nevertheless, the advantage of this study is that by analyzing about 20% of the entire elderly population data, it could represent the entire geriatric population of Korea. In addition, we also performed systematic analyses regarding the impact of the safety warning on domperidone prescriptions, including patient comorbidity, co-medications, prescription dose and duration, and ECG monitoring. Although we only compared the absolute numbers of relative risk for the two years, we confirmed that positive changes towards safe domperidone use occurred.

## 5. Conclusions

In conclusion, after the 2014 regulatory action was issued, domperidone was prescribed more safely in elderly patients, including decreases in prescription frequency, maximum daily dose, and duration of continuous prescription. Thus, the implementation of Korean health regulatory policies and/or system can promote safer prescription practices.

## Figures and Tables

**Table 1:** The characteristics of patients prescribed domperidone in 2011 and 2016.

Characteristics of Patients		No. of Patients (%)		OR (95% CI)
		2011	2016	
Domperidone use	Non-user	903,934 (84.2)	1,315,362 (99.1)	Reference
	User	169,249 (15.8)	12,093 (0.9)	0.049 (0.048, 0.050)
Sex	Male	57,402 (33.9)	4417 (36.5)	Reference
	Female	111,847 (66.1)	7676 (63.5)	0.89 (0.86, 0.93)
Age group	≤65 and <70	57,299 (33.9)	2865 (23.7)	Reference
	≤70 and <75	51,959 (30.7)	2985 (24.7)	1.15 (1.09, 1.21)
	≤75	59,991 (35.4)	6243 (51.6)	2.08 (1.99, 2.18)
Insurance type	Health insurance	154,471 (91.3)	10,592 (87.6)	Reference
	Medical aid	14,536 (8.6)	1468 (12.1)	1.47 (1.39, 1.56)
	Veterans	242 (0.1)	33 (0.3)	1.99 (1.38, 2.86)

**Table 2:** The safety characteristics of domperidone prescriptions in 2011 and 2016.

Characteristics of Patients		No. of Patients/Prescription (%)		OR (95% CI)
		2011	2016	
Number of comorbid diseases ^a^	0	108,368 (64.0)	8363 (69.2)	Reference
	1	47,614 (28.1)	3268 (27.0)	0.89 (0.85, 0.93)
	2	12,013 (7.1)	429 (3.5)	0.46 (0.42, 0.51)
	3	1203 (0.7)	33 (0.3)	0.36 (0.25, 0.50)
	4	51 (<0.1)	0 (0.0)	0.00 (0.00, 0.00)
Type of comorbid disease	Hepatic disease	47,900 (28.3)	1683 (13.9)	0.41 (0.39, 0.43)
	Renal disease	15,181 (9.0)	871 (7.2)	0.79 (0.73, 0.85)
	Cardiac arrhythmia	6489 (3.8)	195 (1.6)	0.41 (0.36, 0.48)
	Parkinson’s disease	5883 (3.5)	1476 (12.2)	3.86 (3.63, 4.10)
Maximum dose of domperidone in each prescriptions ^b^	≤30 mg/day	550,690 (91.2)	24,423 (99.2)	Reference
	>30 and ≤40 mg/day	11,556 (1.9)	98 (0.4)	0.19 (0.16, 0.23)
	>40 mg/day	41,716 (6.9)	102 (0.4)	0.06 (0.05, 0.07)
Maximum continuous duration of prescribed domperidone ^a^	≤7 days	104,824 (61.9)	6872 (56.8)	Reference
	>7 days	64,425 (38.1)	5221 (43.2)	1.24 (1.19, 1.28)

^a^ The percentage was calculated for total number of patients (169,249 in 2011 and 12,093 in 2016) prescribed domperidone at least once; ^b^ The percentage was calculated for total number of prescription of domperidone (603,962 in 2011 and 24,623 in 2016).

**Table 3:** The safety characteristics of patients prescribed domperidone in 2011 and 2016.

Characteristics of Patients		No. of Prescriptions	
		2011 (N = 603,962)	2016 (N = 24,623)
Number of co-prescribing medications	0	450,217	13,959
	1	141,383	7631
	2	11,791	2179
	≥3	571	854

**Table 4:** Co-prescribed medications may increase cardiac side effects due to interacting with domperidone.

Drug Category (ATC ^a^ Code)	No. of Prescriptions	
	2011 (N = 603,962)	2016 (N = 24,623)
Drugs for acid related disorders (A02)	96,031	2811
Diuretics (C03)	28,225	4085
Antibacterials for systemic use (J01)	17,080	1112
Psychoanaleptics (N06)	16,143	2470
Antihistamines for systemic use (R06)	8765	143
Antimycotics for systemic use (J02)	4214	156
Calcium channel blockers (C08)	3669	963
Antithrombotic agents (B01)	3478	594
Psycholeptics (N05)	2853	2145
Drugs for obstructive airway disease (R03)	2478	150
Miscellaneous	341	67
Drugs for cardiac disease (C01)	292	160
Analgesics (N02)	92	6
Antivirals for systemic use (J05)	0	0

^a^ ATC: Anatomical Therapeutic Chemical Classification System.

## Supplementary Materials

The following are available online at https://www.mdpi.com/1660-4601/16/16/2985/s1. Table S1: List of medications may increase cardiac side effects due to interacting with domperidone.
