# Supplementary material for: Impact of Safety Warning on Domperidone Prescribing for Geriatric Patients in South Korea: Analysis of National Insurance Claim Data
Source: Int J Environ Res Public Health. 2019 Aug 20;16(16):2985. doi: 10.3390/ijerph16162985 (PMC6719182; doi:10.3390/ijerph16162985)
Supplement: Supplementary file 1 [file ijerph-16-02985-s001.pdf]

Supplement Table S1. List of medications may increase cardiac side effects due to interacting with domperidone

Drug category	Medication
Drugs for acid related disorders (A02)	cimetidine, cisapride
Antithrombotic agents (B01)	cilostazol, warfarin
Drugs for cardiac disease (C01)	flecainide, amiodarone
Diuretics (C03)	bumetanide, furosemide, hydrochlorothiazide, indapamide, torasemide
Calcium channel blockers (C08)	nicardipine, isradipine, verapamil, diltiazem
Antibacterials for systemic use (J01)	erythromycin, clarithromycin, azithromycin, levofloxacin, norfloxacin, ofloxacin
Antimycotics for systemic use (J02)	fluconazole, itraconazole, ketoconazole
Antivirals for systemic use (J05)	indinavir, ritonavir, nelfinavir
Analgesics (N02)	naratriptan, sumatriptan, zolmitriptan
Psycholeptics (N05)	chlorpromazine, haloperidol, perphenazine, quetiapine, risperidone, ziprasidone
Psychoanaleptics (N06)	amoxapine, trazodone, venlafaxine, fluvoxamine, paroxetine, fluoxetine, sertraline, amitriptyline, imipramine
Drugs for obstructive airway disease (R03)	formoterol, salmeterol, zafirlukast
Antihistamines for systemic use (R06)	azelastine, clemastine
Miscellaneous	tamoxifen, phenytoin, sildenafil, octreotide, pentamidine, tizanidine
